# Rapid and Efficient Cell-to-Cell Transmission of Avian Influenza H5N1 Virus in MDCK Cells Is Achieved by Trogocytosis

**DOI:** 10.3390/pathogens10040483

**Published:** 2021-04-16

**Authors:** Supasek Kongsomros, Suwimon Manopwisedjaroen, Jarinya Chaopreecha, Sheng-Fan Wang, Suparerk Borwornpinyo, Arunee Thitithanyanont

**Affiliations:** 1 Department of Microbiology, Faculty of Science, Mahidol University, Bangkok 10400, Thailand; supasek.kon@student.mahidol.ac.th (S.K.); swiboonut@gmail.com (S.M.); jarinya.cha@student.mahidol.ac.th (J.C.); 2 Department of Medical Laboratory Sciences and Biotechnology, College of Health Sciences, Kaohsiung Medical University, Kaohsiung 80708, Taiwan; wasf1234@kmu.edu.tw; 3 Excellence Center for Drug Discovery (ECDD), Faculty of Science, Mahidol University, Bangkok 10400, Thailand; suparerk.bor@mahidol.ac.th; 4 Department of Biotechnology, Faculty of Science, Mahidol University, Bangkok 10400, Thailand

**Keywords:** cell-to-cell transmission, H5N1, trogocytosis

## Abstract

Viruses have developed direct cell-to-cell transfer strategies to enter target cells without being released to escape host immune responses and antiviral treatments. These strategies are more rapid and efficient than transmission through indirect mechanisms of viral infection between cells. Here, we demonstrate that an H5N1 influenza virus can spread via direct cell-to-cell transfer in Madin-Darby canine kidney (MDCK) cells. We compared cell-to-cell transmission of the H5N1 virus to that of a human influenza H1N1 virus. The H5N1 virus has been found to spread to recipient cells faster than the human influenza H1N1 virus. Additionally, we showed that plasma membrane exchange (trogocytosis) occurs between co-cultured infected donor cells and uninfected recipient cells early point, allowing the intercellular transfer of viral material to recipient cells. Notably, the H5N1 virus induced higher trogocytosis levels than the H1N1 virus, which could explain the faster cell-to-cell transmission rate of H5N1. Importantly, this phenomenon was also observed in A549 human lung epithelial cells, which are representative cells in the natural infection site. Altogether, our results provide evidence demonstrating that trogocytosis could be the additional mechanism utilized by the H5N1 virus for rapid and efficient cell-to-cell transmission.

## 1. Introduction

Viral infection occurs when viruses enter target cells and begin to multiply. New viral particles released from the infected producer cells are then transmitted to the next target cells. The classical transmission pathway involves the release of the infectious virion into the extracellular fluid for further spread to new target cells via a cell-free infection mechanism [1]. It is the best mechanism for infecting across long distances and spreading to new hosts [1]. Through the co-evolution of viruses with their host, viruses have developed means for overcoming host barriers and evading host immune defenses, including neutralizing antibodies and antiviral restriction factors. One such evasion tactic involves developing alternative modes of transmission, specifically, cell-to-cell viral transfer, in which the virus can hijack cell–cell contact junctions and use them for transmitting viral material to the target cell bypassing diffusion of the virus into the extracellular fluid [1,2,3,4].

Cell-to-cell transmission has been previously reported for many viruses, which adopt various means of spreading [1,2,3,4]. The neurotropic viruses, such as herpesvirus and rhabdovirus, were able to directly spread to another cell across a neuron network via neurological synapses [2,3]. The retroviruses, including human immunodeficiency virus 1 (HIV-1) and human T-cell lymphotropic virus type 1 (HTLV-1), can create a transient contact, termed virological synapse, to promote viral spread between immune cells [5,6]. Additionally, retroviruses have been reported that utilize actin-containing structures, tunneling nanotubes (TNT), or filopodium, existing between cells to directly transmit to the target cell [5,6,7]. The use of occludin and claudin-1 for viral transfer was documented for the cell–cell transmission of hepatitis C virus (HCV) between hepatocytes at tight junctions [8,9]. Moreover, plasma membrane exchange (trogocytosis) between cells has been reported to be an additional mechanism involved in cell-to-cell transmission. Trogocytosis is known as a mechanism of intercellular communication between immune cells, by which they can acquire the membrane components from partner cells, leading to alteration of their functional capabilities [10]. Transfer via trogocytosis is fast, occurring within a few minutes [11]. During this process, two conjugated cells’ membranes are briefly fused, and surface molecules are transferred to another cell [11,12]. Membrane fusion during trogocytosis also leads to the exchange of cytosolic materials with partner cells [12]. This mechanism was found to be exploited by intracellular bacteria to directly transfer to neighboring cells [12]. HIV also has been reported to use trogocytosis to transfer viral RNA from infected T cells to uninfected T cells, thereby promoting infection [13,14]. Furthermore, the formation of bridged membrane structures during trogocytosis was shown to help HIV virions surf extracellularly towards the uninfected cell [13,14].

There have been reports that the human influenza A virus can spread to target cells without particle release, allowing it to evade neutralizing antibodies and neuraminidase inhibitors [4,15]. The use of a tunneling nanotube was previously identified as the mechanism of this cell-to-cell transfer, which facilitates infection of neighboring cells with the human influenza A virus [16,17]. Unlike the human influenza virus, avian influenza H5N1 viruses can disseminate from the respiratory tract to other organs leading to the induction of high viral load and cytokine storm [18]. The H5N1 virus in humans also exhibits faster rates of infection and replication to higher titers compared to the human influenza H1N1 virus [19]. Thus, it is possible that the H5N1 virus can undergo cell-to-cell transmission more efficiently than the human influenza virus to disseminate from the respiratory system, evade immune responses, and spread rapidly to result in a highly virulent infection. However, cell-associated transmission by H5N1 has not yet been explored. Thus, this study aimed to investigate the cell-to-cell transmission of the H5N1 virus and to determine whether the H5N1 virus uses this means more efficiently than the H1N1 virus. Here, we show evidence of cell-to-cell transmission of the H5N1 virus in Madin-Darby canine kidney (MDCK) cells and demonstrate that cell-to-cell transmission of H5N1 occurred faster than that of the H1N1 virus at early time points of infection. Interestingly, we found that trogocytosis occurs between the influenza virus-infected donor cells and recipient cells during co-cultures. Our data showed that nucleoproteins were directly transferred to recipient cells through trogocytosis. Moreover, we performed the experiments in A549 human lung epithelial cells to assess this phenomenon, and it was demonstrated in these cell lines as well. As the trogocytosis process occurred very fast for H5N1 compared to H1N1, the high efficiency of induction of trogocytosis could be an important factor contributing to the observed differences in infection rate between the influenza virus subtypes. We propose that trogocytosis might be an important additional mechanism used in cell-to-cell transmission of the H5N1 virus.

## 2. Results

### 2.1. Cell-to-Cell Transmission of Avian Influenza H5N1 Virus

**Figure 1:**
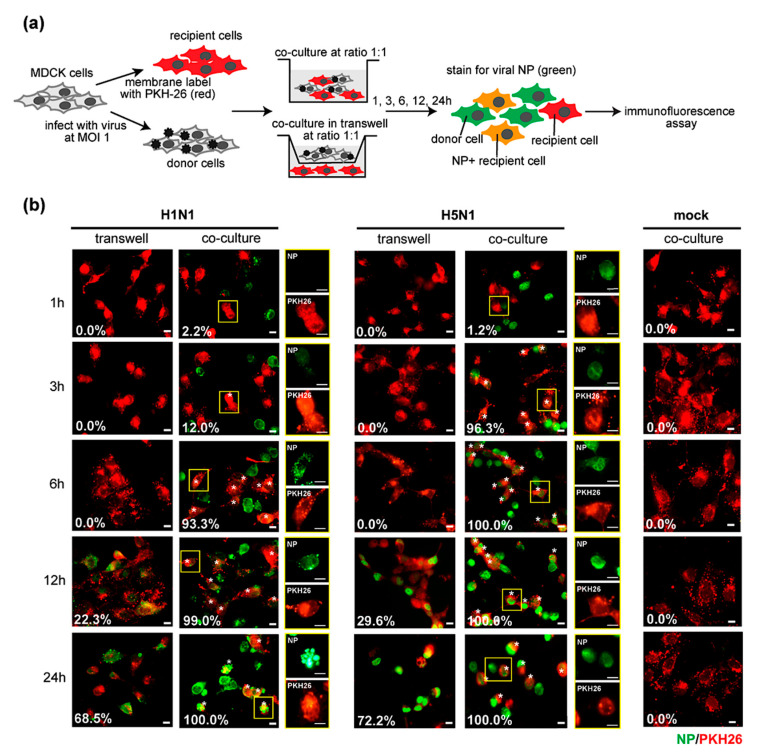
Cell-to-cell transmission of influenza A virus. (**a**) Madin-Darby canine kidney (MDCK) cells were infected with H5N1 or H1N1 viruses at a multiplicity of infection (MOI) of 1 for 8 h. The infected MDCK cells were used as donor cells. Donor cells were directly co-cultured or indirectly cultured in a Transwell with the PKH26-labeled MDCK cells (recipient cells) at a ratio of 1:1 for 1, 3, 6, 12, 24 h. Cells were analyzed by immunofluorescence microscopy after staining with anti- nucleoproteins (NP). Mock-infection was performed in parallel as a negative control. Viral NP expression (green) in recipient cells (red) in co-cultures and Transwells at indicated time points are shown in (**b**). The asterisk represents the NP+ recipient cells in the co-cultures. The percentages of NP+ recipient cells in each condition were quantified by manual counting indicated as a white number at the lower left. The data are presented as the mean of three independent experiments. The squared regions are shown as a single NP or PKH26 signal. Scale bar: 10 µm.

Although some evidence of cell-to-cell transmission has been reported in human influenza viruses [15,16,17], this transmission mode has not been yet studied in the avian influenza H5N1 virus. To demonstrate cell-to-cell transmission in the H5N1 virus, co-culture and Transwell assays were performed. MDCK cells were infected with the H5N1 virus at a multiplicity of infection (MOI) of 1 for 8 h used as donor cells. PKH26 pre-labeled MDCK cells were used as recipient cells. Infected donor cells were directly co-cultured with or indirectly co-cultured in a Transwell with recipient cells at a ratio of 1:1 for 1, 3, 6, 12, and 24 h. Cells were then subjected to fluorescence microscopy after staining with FITC conjugated anti-NP (Figure 1a). Human influenza H1N1 virus (PR8 strain) and mock-infected cells were used as controls. The number of NP+ recipient cells under each condition was quantified by manual counting and indicated as a white number at the lower left of each image. The fluorescence imaging showed that the infected recipient cells, as assessed by intracellular nucleoproteins (NP) expression, were initially detected in H5N1 and H1N1-infected co-cultures after 3 h of co-culture, while the recipient cells in the Transwells became infected after 12 h of co-culture (Figure 1b). These results indicated that cell–cell contact enhanced the rate of H5N1 and H1N1 transmission. The results suggest that cell–cell contact is required for influenza virus transmission.

### 2.2. Cell-to-Cell Transmission Rate of H5N1 Virus Is Faster Than H1N1 Virus

**Figure 2:**
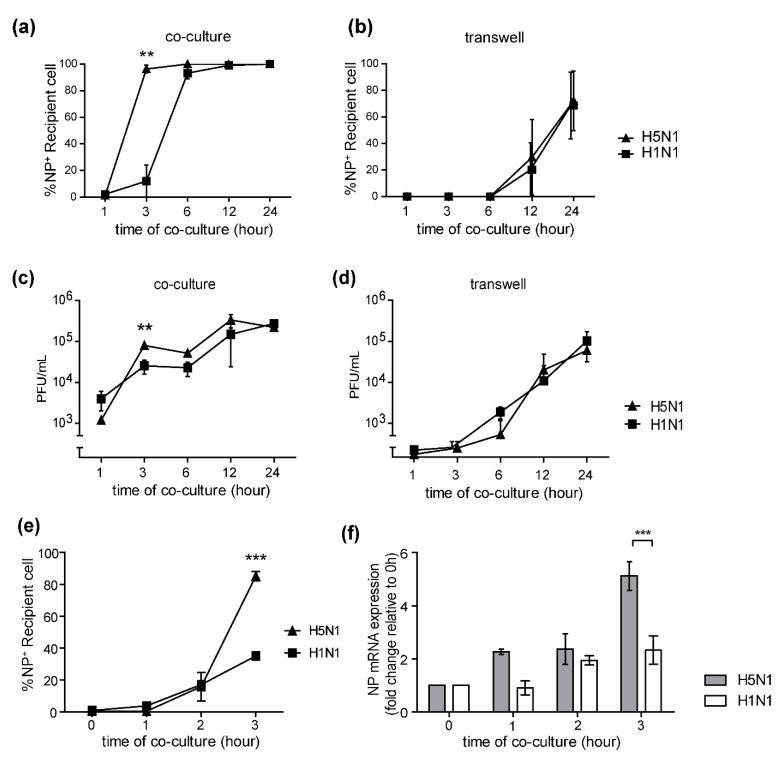
H5N1 cell-to-cell transmission is faster than H1N1. (**a**,**b**) A comparison of the infection rate of H1N1 and H5N1 viruses in co-cultures and Transwells by manual counting of the NP+ recipient cells from Figure 1b were shown. The culture supernatants of co-cultures at 1, 3, 6, 12, and 24 h were collected, and levels of virus production were measured by plaque assay. (**c**,**d**) Kinetics of viral titer growth of H5N1 and H1N1 viruses in co-cultures and Transwells at the indicated time is shown. (**e**) A comparison of NP+ recipient cells in the co-cultures with H5N1 and H1N1 infection at the early time point by flow cytometry is shown. Statistical analysis was performed using unpaired *t*-test. A co-culture assay was performed. (**f**) NP gene expression was determined by qPCR. The fold change of NP expression levels of H5N1 and H1N1-infected co-cultures was shown. The data are presented as the mean ± SEM of three independent experiments. Statistical analysis was performed using one-way ANOVA. ** *p* < 0.005, *** *p* < 0.001.

High viral loads and systemic viral dissemination in the H5N1 infection led us to determine whether the H5N1 virus utilized the cell-to-cell transmission mechanism more efficiently than the human influenza virus. We compared the number of NP+ recipient cells in the H5N1 and H1N1-infected co-cultures and Transwells from Figure 1b. The results showed that the NP+ recipient cells in the H5N1-infected co-cultures were significantly higher than that of H1N1 at 3 h of co-culture (Figure 2a), while there was no statistically significant difference between the number of NP+ recipient cells in the H5N1 and H1N1-infected Transwells (Figure 2b). The amount of virus in the culture supernatant was also determined by plaque assay. The viral production in the H5N1-infected co-culture was significantly higher than that of H1N1 after 3 h of co-culture (Figure 2c), but no difference in viral production between the H5N1 and H1N1 viruses was observed in-infected Transwells (Figure 2d). We further quantified the efficiency of viral transmission in co-cultures at early time points (1–3 h of co-culture) by flow cytometry. The results showed that there were significantly higher numbers of NP+ recipient cells in the H5N1-infected co-cultures than in H1N1-infected co-cultures after 3 h of co-culture (Figure 2e), which was confirmed by quantification by immunofluorescence imaging (Figure 1b). The NP gene expression levels in infected co-cultures at the early time point were also determined by qPCR. The fold change in NP gene expression was analyzed using the delta-delta Ct method, relative to the expression level at the t = 0 timepoint normalized to β-actin as the reference gene. The NP gene expression levels in the co-culture experiments for H5N1 and H1N1 infections were compared (Figure 2f). The results showed that the fold change in NP expression in the H5N1-infected co-cultures was significantly higher than that of H1N1 after 3 h of co-culture (Figure 2f). Altogether, our results conclude that the cell-to-cell transmission rate of the H5N1 virus is faster than that of the H1N1 virus.

### 2.3. H5N1 Virus Induces Higher Levels of Trogocytosis Than H1N1 Virus

**Figure 3:**
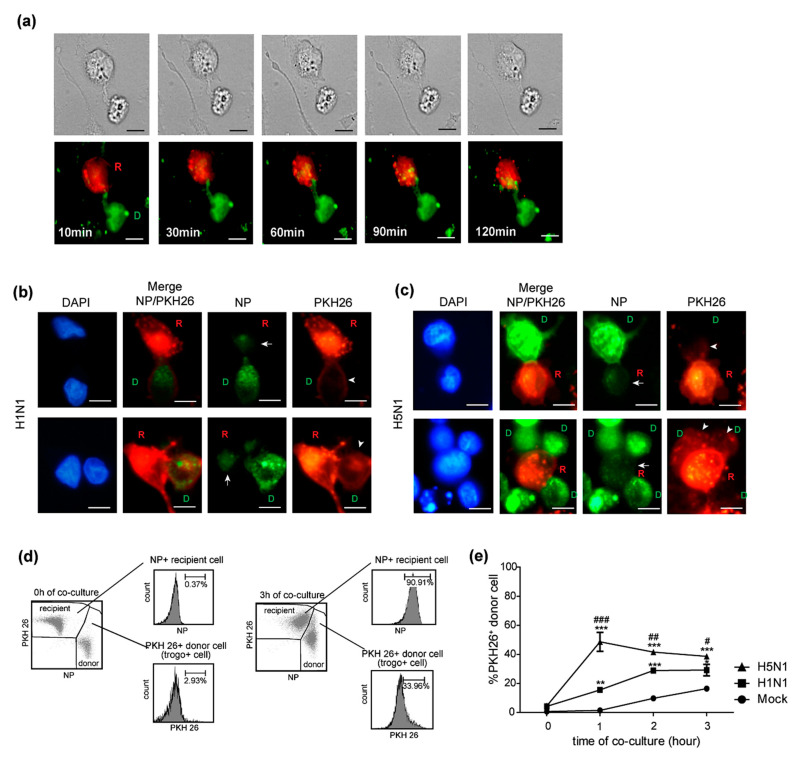
H5N1 virus induces higher levels of trogocytosis than the H1N1 virus. MDCK cells were infected with H5N1 at an MOI of 1 for 8 h. Infected MDCK cells labeled with PKH67 were used as donor cells. Donor and PKH26-labeled recipient cells were directly co-cultured. Cells were then imaged every 10 min using live-cell microscopy. (**a**) Live-cell images showed that membrane exchange occurred between the infected donor and recipient cells. Co-culture assay of H5N1 and H1N1 infection was performed following Figure 1a–c At early time points (1–3 h of co-culture), the donor cell, outlined by the green fluorescent labeled NP, transfers NP to recipient cells (red) as indicated by the arrow in the NP panel. Transfer of the PKH26-labeled membrane of recipient cells to donor cells (trogocytosis) was observed, as indicated by the arrowhead (PKH26 panel in (**b**,**c**). The membrane exchange (trogocytosis) between donor cells and recipient cells was analyzed by flow cytometry, which was quantified by measuring the percentage of plasma membrane exchange in infected donor cells (PKH26+ donor cells). (**d**) Gating strategies were shown. (**e**) The percentages of the PKH26+ donor cells in H5N1, H1N1, and mock-infected co-culture at the indicated time points are summarized. The data are presented as the mean ± SEM of three independent experiments. Statistical analysis was performed using two-way ANOVA. * *p* < 0.05, ** *p* < 0.005, *** *p* < 0.001 for the H5N1 and H1N1 viruses vs. mock. # *p* < 0.05, ## *p* < 0.005, ### *p* < 0.001 for the H5N1 vs. H1N1 viruses. Scale bars: 10 µm.

The rapid spreading of the H5N1 virus within a period of time as short as 2–3 h spurred us to explore the mechanisms involved in such H5N1 cell-to-cell transmission. Trogocytosis is a mechanism in which the plasma membrane and its associated molecules are exchanged between conjugated immune cells. A key characteristic of trogocytosis is that it is fast, occurring within a minute [11]. Besides immune cells, trogocytosis was documented in cells involved in adherence, such as between cancer cells and stromal cells [20,21]. Furthermore, trogocytosis has also been reported to play a role in cell-to-cell transmission of HIV [13,14]. Thus, we hypothesized that the rapid cell-to-cell transmission of the H5N1 virus might be explained by trogocytosis in infected co-cultures. Live-cell imaging was performed to study trogocytosis between and PKH67 (green)-labeled H5N1-infected donor and PKH26 (red)-labeled recipient cells. The results demonstrated evidence of trogocytosis, with the membrane of the donor cells (D) being transferred into the recipient cells (R) initiating within 10 min of co-culture (Figure 3a). Infected donor cells were then directly co-cultured with the PKH26-labeled recipient cells for 0, 1, 2, and 3 h. Cells were then stained for NP and analyzed by fluorescence microscopy. The fluorescent images demonstrated that the PKH26-labeled components of the membrane of recipient (R) cells were detected in donor cells (D), as indicated by an arrowhead in the PKH26 panel in both in H1N1 and H5N1-infected co-cultures, as shown in Figure 3b,c, respectively, which indicated plasma membrane exchange between donor and recipient cells. Viral NPs from infected donor cells were also transferred to the recipient cells at the same time, as indicated by the arrow in the NP panels (Figure 3b,c). The results implied that trogocytosis occurred between infected donor cells and recipient cells at the early time point of co-culture, which facilitated the viral protein transfer. The differences in cell-to-cell transmission between the H5N1 and H1N1 viruses motivated us to determine the efficiency of trogocytosis induction by these two viruses. H5N1 and H1N1-infected cells were co-cultured with recipient cells for 1, 2, and 3 h. Cells were then harvested and analyzed by flow cytometry after staining with FITC conjugated anti-NP. Recipient and donor cells were gated, and trogocytosis was quantified by measuring the percentage of PKH 26+ donor cells (trogo+ cells) as described in Figure 3d. The percentages of trogo+ cells in the H5N1-infected co-cultures were significantly higher than that in H1N1 virus-infected cultures at 1, 2, and 3 h (Figure 3e). The results revealed that trogocytosis induced by H5N1 was greater than that induced by H1N1. We suggest that trogocytosis may play an important role in cell-to-cell transmission of the influenza virus at early time points, which is clearly correlated with the rapid rate of cell-to-cell transmission.

### 2.4. Tunneling Nanotubes (TNTs) Are Formed between Donor and Recipient Cells after Trogocytosis

**Figure 4:**
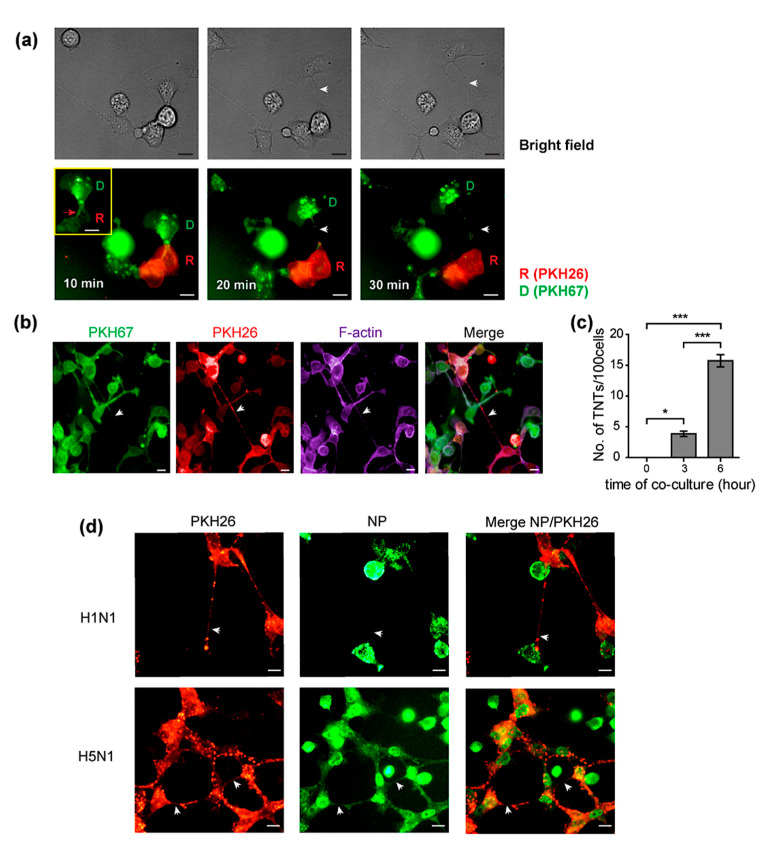
Tunneling nanotubes (TNTs) are formed between donor and recipient cells after trogocytosis. MDCK cells were infected with H5N1 at an MOI of 1 for 8 h. Infected MDCK cells labeled with PKH67 were used as donor cells. Donor and PKH26-labeled recipient cells were directly co-cultured. Cells were then imaged every 10 min using live-cell microscopy. Live cell images showed the TNT formation (indicated as white arrow) between the infected donor and recipient cells after trogocytosis ((**a**), middle and right panels). The red box shows the PKH67-labeled membrane of donor cells transferred to recipient cells (trogocytosis) indicated as a red arrow ((**a**), left panel). The co-cultures were fixed and stained for F-actin. (**b**) The fluorescence images showed the presence of PKH67, PKH26, and F-actin on the TNT indicated as an arrow. (**c**) The TNTs were then quantified at the indicated time point. Co-culture assays of H5N1 and H1N1 infection were performed following Figure 1a. (**d**) The presence of tunneling nanotubes (arrow) was demonstrated in both H5N1 and H1N1-infected co-cultures. (**d**) NP existed in the TNT as indicated by the arrow in the NP panel. The data are presented as the mean ± SEM of three independent experiments. Statistical analysis was performed using one-way ANOVA * *p* < 0.05, *** *p* < 0.001. Scale bars: 10 µm.

Previously, the human influenza virus was reported to exploit actin-containing tunneling nanotubes for cell-to-cell spread, by which detectable levels of viral mRNA were observed in target cells at 6 h [17]. In our hands, we found that trogocytosis appeared between donor and recipient cells at an earlier time point of co-culture than the mechanism involving TNTs. We investigated whether TNTs occurred during the observed process of trogocytosis. Live-cell images of PKH67 pre-labeled H5N1-infected donor cells co-cultured with the PKH26 recipient cells showed that TNTs were formed when the cells moved apart after cell–cell contact during trogocytosis (Figure 4a). The co-cultures were then fixed and stained for F-actin with Alexa 647 phalloidin. The results showed that both PKH26 and PKH67 membrane labeling dyes were found within the TNTs between donor and recipient cells (Figure 4b), indicating that TNTs were formed by plasma membranes of both cells, suggesting that an exchange of membrane might occur between donor and recipient cells. In addition to the presence of a dual labeled membrane, F-actin was also presented inside TNTs (Figure 4b). Moreover, the number of TNTs/100 cells in the H5N1-infected co-culture was then quantified by manual counting. We found that the number of TNTs was significantly increased over time after 1 h of co-culture (Figure 4c). Representative fluorescent images of the H5N1 and H1N1-infected co-cultures at 6 h demonstrated that TNTs were formed between the cells and contained viral NP, indicating that TNTs provided a route for the viral protein transfer at a later time point (Figure 4d). These findings suggested that after cell–cell contact through trogocytosis, the two cells formed thin membrane TNTs when the cells separated. Hence, the direct transmission process between the cells could continue after disassembly of the trogocytosis junction.

### 2.5. Cell-to-Cell Transmission of H5N1 Virus Requires Actin Polymerization

**Figure 5:**
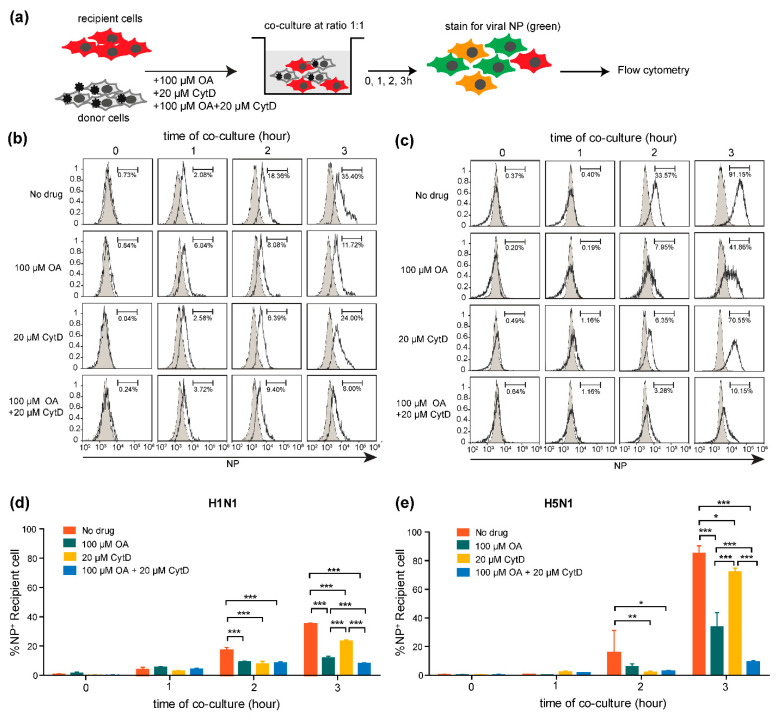
Influenza A virus cell-to-cell transmission requires actin polymerization. (**a**) MDCK cells were infected with H5N1 or H1N1 viruses at an MOI of 1 for 8 h. The infected MDCK cells were used as donor cells. Donor and PKH26-labeled recipient cells were directly co-cultured in the presence of 100 µM oseltamivir (NA inhibitor) or 20 µM cytochalasin D, or with a combination of 100 µM oseltamivir and 20 µM cytochalasin D for 1, 2 and 3 h. Cells were then analyzed by flow cytometry after staining with anti-NP. The results are shown in (**b**,**c**) for H1N1 and H5N1 viruses, respectively. Data are representative of 3 independent experiments. The percentages of NP+ recipient cells are summarized in (**d**,**e**). The data are presented as the mean ± SEM of three independent experiments. Statistical analysis was performed using two-way ANOVA. * *p* < 0.05, ** *p* < 0.005, *** *p* < 0.001.

Previously, we reported that cell–cell contact was required for the efficient transmission of the influenza virus. It was also reported that treatment with cytochalasin D, a specific inhibitor of actin polymerization, disrupted cell–cell interactions, resulting in attenuated cell–cell spreading of the virus [16]. To examine whether H5N1 cell-to-cell transmission requires actin polymerization, co-culture assays were performed in the presence of oseltamivir (NA inhibitor), cytochalasin D, and the combination of oseltamivir and cytochalasin D. Infected donor cells were co-cultured with the PKH26 pre-labeled recipient cells in the presence of indicated drugs for 0, 1, 2, 3 h. Cells were then harvested and subjected to flow cytometry after staining with FITC conjugated anti-NP (Figure 5a). The histograms of NP+ recipient cells in infected co-cultures (open histogram) compared to mock-infected co-cultures (filled histogram) of each condition at the indicated time points are shown in Figure 5b,c for H1N1 and H5N1 infection, respectively. Our results showed that both H5N1 and H1N1 viruses could spread to the recipient cells despite the presence of oseltamivir (Figure 5d,e), indicating that treatment of the co-culture with oseltamivir efficiently prevented cell-free infection; therefore, the virus can shift itself to spread via a cell-to-cell transmission mode. We also observed that the NP+ recipient cells were immediately decreased in H1N1 and H5N1 co-cultures when treated with cytochalasin D alone (Figure 5d,e), but were even further decreased in combination treatment with oseltamivir and cytochalasin D at 2 and 3 h of co-culture, which blocked both cell-free transmission and actin polymerization (Figure 5d,e) than no drug treatments. The results indicate that H5N1 cell-to-cell transmission required actin polymerization. Altogether, we propose that cell-to-cell transmission of the H5N1 virus is an actin-dependent pathway, which may provide an important route for promoting resistance to antiviral treatments.

### 2.6. Trogocytosis in A549 Human Lung Epithelial Cell Co-Cultures Is Associated with the Infection Rate of H5N1 Virus

**Figure 6:**
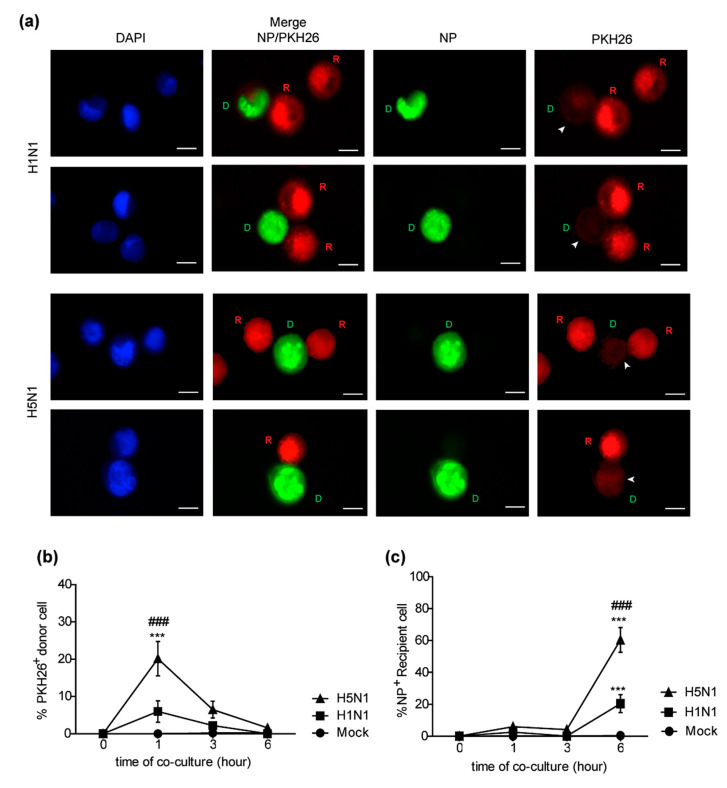
Trogocytosis in the H5N1-infected A549 co-cultures is correlated with the infection rate of the H5N1 virus. A549 cells were infected with the H5N1 or H1N1 viruses at an MOI of 10 for 24 h. The infected donor cells were directly co-cultured with the PKH26-labeled A549 cells (recipient cells) at a ratio of 1:1 for 0, 1, 3 and 6 h. Cells were then analyzed by immunofluorescence microscopy and flow cytometry after NP staining. (**a**) Transfer of the PKH26-labeled membrane of recipient cells (R) to donor cells (D) was observed, as indicated by the arrowhead in the PKH26 panel. Trogocytosis between donor cells and recipient cells was analyzed by flow cytometry, which was quantified by measuring the percentage of plasma membrane exchange in infected donor cells (PKH26+ donor cells). Gating strategies were performed as followed Figure 3d. (**b**) The percentages of the PKH26+ donor cells in the H5N1, H1N1, and mock-infected co-cultures at the indicated time points are summarized. (**c**) The percentages of NP+ recipient cells in each condition are shown. The data are presented as the mean ± SEM of three independent experiments. Statistical analysis was performed using two-way ANOVA. *** *p* < 0.001 for the H5N1 and H1N1 viruses vs. mock. ### *p* < 0.001 for the H5N1 vs. H1N1 virus. Scale bars: 10 µM.

We previously demonstrated the occurrence of trogocytosis in co-cultures of MDCK cells contributing to the rapid viral transmission of the H5N1 virus. Here, we examined whether the same occurred in A549 human lung epithelial cells that may be representative cells at the physiological site of influenza virus infection. To demonstrate the event of trogocytosis in A549 cells, A549-infected donor cells were directly co-cultured with the PKH26 labeled A549 recipient cells for 0, 1, 3, and 6 h. Cells were then stained for NP and analyzed by fluorescence microscopy. The fluorescent images demonstrated that the PKH26-labeled membrane of recipient (R) cells was detected in donor cells (D), as indicated by an arrowhead in the PKH26 panel in both H1N1 and H5N1-infected co-cultures (Figure 6a). The results confirmed that trogocytosis occurred between A549-infected donor cells and recipient cells. We next examined the efficiency of trogocytosis induction by the H5N1 and H1N1 viruses and the rate of viral transmission of these two viruses in A549 cells. H5N1 and H1N1-infected A549 donor cells were co-cultured with the PKH26-labeled A549 recipient cells at the ratio 1:1 for 0, 1, 3 and 6 h. Cells were then harvested and analyzed by flow cytometry after staining with FITC conjugated anti-NP. A549 recipient and donor cells were gated, and trogocytosis was quantified by measuring the percentage of PKH 26+ donor cells (trogo+ cells) as followed in Figure 3d. The percentages of trogo+ cells in the H5N1-infected co-cultures were significantly higher than that in H1N1 virus-infected cultures at 1 h (Figure 6b). Quantification of NP expression on the A549 recipient cells by flow cytometry also showed that the percentage of NP + A549 recipient cells was significantly higher in the H5N1-infected co-cultures compared to those in H1N1-infected co-cultures co-culture at 6 h (Figure 6c). Our results revealed that the higher trogocytosis levels in H5N1 are correlated with the higher rate of H5N1 infection in the infected co-cultures suggesting that trogocytosis could also play a role during H5N1 infection in A549 human lung epithelial cells.

## 3. Discussion

Avian influenza H5N1 virus remains a threat to humans. Although antiviral drugs are currently available for virus treatment and outbreak control, cell-to-cell transmission is a strategy developed by the human influenza virus to escape from antiviral drugs, such as neuraminidase inhibitors, oseltamivir and zanamivir [15,16,22]. According to HIV studies, the cell-to-cell transmission was proposed as the mechanism by which very efficient viral infection of higher orders of magnitude could occur with much shorter viral generation times than cell-free transmission, contributing to rapid infection [23]. Moreover, the efficiency of HIV cell-to-cell spread was found to be related to multiple infections per cell, leading to a massive infection [24]. The high replication rate of the H5N1 virus led us to determine whether it resulted from a level of cell-to-cell transmission. We demonstrated the presence of cell-to-cell transmission of the H5N1 virus and compared the cell-to-cell transmission characteristics between the H5N1 and H1N1 viruses (Figure 1b). The results showed that the H5N1 cell-to-cell transmission was faster than that of the H1N1 virus. Quantification of NP expression on the recipient cells by immunofluorescence assay and flow cytometry showed that the H5N1 NP was expressed on up to 100% of recipient cells in co-cultures after 3 h, while approximately only 20% of recipient cells expressed NP in H1N1-infected co-cultures (Figure 2a,e). The NP expression on the recipient cells of the H5N1 and H1N1 virus was not different in Transwells (Figure 2b). The NP gene expression in the H5N1-infected co-cultures was also significantly higher than that of H1N1 (Figure 2f). We also determined virus production by plaque assay and found that the viral titer of the H5N1 virus was significantly higher than H1N1 at 3 h of co-culture, while there was no difference in the Transwell cultures (Figure 2c,d). Thus, our results confirmed that the H5N1 virus utilized the cell-to-cell transmission pathway more efficiently than the H1N1 virus. However, infectious particles around 10^3^ pfu/mL were detected in the co-culture supernatant, but only around 10^1^ pfu/mL were detected in Transwells (Figure 2c,d). Without detecting nucleoprotein in recipient cells, the co-cultures supernatant’s virions were likely from the infected donor cells. The mechanisms that underline this phenomenon in this study are still unclear. Previous reports demonstrated that cell–cell contact increases the kinetics of viral infection by directing virus assembly towards and budding at cell–cell contact sites in HIV and other viruses [25,26,27,28,29]. In this situation, cell–cell contact may facilitate the kinetic of infection, resulting in the enhanced kinetics of viral assembly and release, as mentioned earlier. Nevertheless, further investigation is needed.

The rapid cell-to-cell transmission observed for H5N1 motivated us to explore the mechanisms used by the H5N1 viruses to achieve such virulence. Trogocytosis was proposed because this mechanism is involved in cell–cell communication and results in the exchange of surface molecules between the cells. Furthermore, a key characteristic of trogocytosis is that such transfer occurs within a minute [11]. Trogocytosis has been demonstrated to play an important role during virus infection, with NK cells and CD8+ T cells acquiring virus receptors, which they normally do not express, from their target cells, leading to the expression of these receptors on the surface of these cells. This phenomenon subsequently rendered these immune cells susceptible to viral infection [30,31]. Our team also has found that B cells acquire an α2,3 SA receptor from monocytes via cell contact-dependent trogocytosis, which subsequently enables the H5N1 virus to infect B cells [32]. Additionally, HIV was reported to use trogocytosis to spread to adjacent cells by directly transferring viral proteins to the conjugated cells [13,14]. In the present study, we showed that trogocytosis occurred between the infected donor and recipient MDCK cells within 10 min of co-culture (Figure 3a). Intracellular viral nucleoproteins (NP) from the infected donor cells were transferred to the recipient cells during membrane exchange, as shown in Figure 3b,c suggesting that trogocytosis led to the brief fusion of the cells, and NPs contained in the cytoplasm were subsequently transferred to the MDCK recipient cells. Furthermore, within 1 h of co-culture, we also demonstrated the evidence of trogocytosis between the co-cultures of infected A549 donor cells and the recipients (Figure 6a). Our findings were supported by previous evidence shown for HIV and intracellular bacteria in which it was shown that membrane fusion during trogocytosis led to the exchange of cytosolic materials with partner cells, contributing to direct transmission to neighbor cells [12,14].

**Figure A1:**
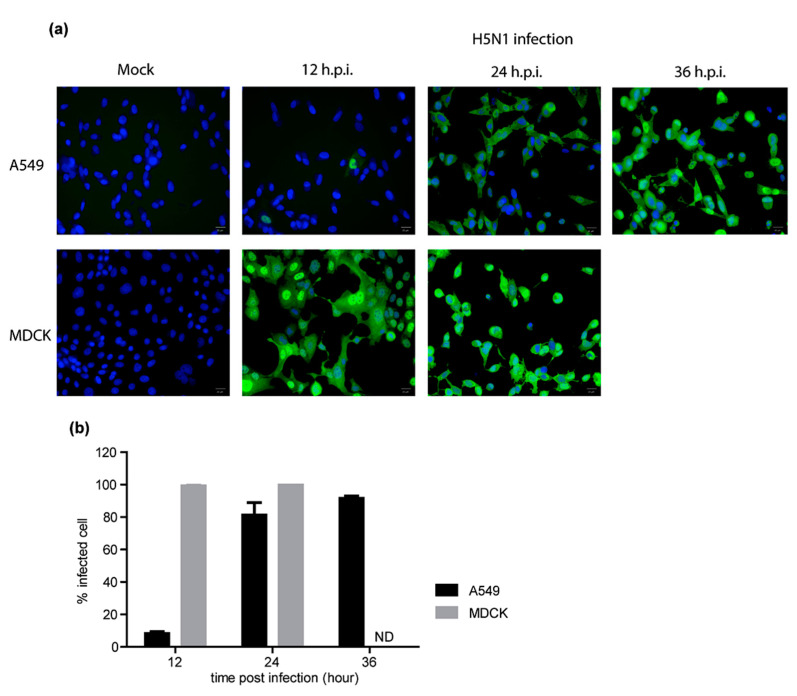
H5N1 infection in A549 and MDCK cells. (**a**) A549 and MDCK cells were infected with H5N1 viruses at an MOI of 1 for 12, 24 and 36 h. Cells were then analyzed by flow cytometry after staining with anti-NP. Mock-infection was performed in parallel as a negative control. (**b**) The percentages of NP+ recipient cells in each condition were quantified by manual counting. The data are presented as the mean of three independent experiments. Scale bars: 20 µM.

The efficiency of trogocytosis induction could be an important factor in dictating the differences in cell-to-cell transmission between influenza subtypes. Hence, we compared the induction of trogocytosis by the H5N1 and H1N1 viruses in MDCK and A549 co-cultures by flow cytometry. We found that the H5N1 virus induced higher levels of trogocytosis than H1N1 in both MDCK cells (Figure 3e) and A549 cells (Figure 6b). The high level of trogocytosis induction by the H5N1 virus correlated with the observed rapid rate of H5N1 viral cell-to-cell transmission in the MDCK (Figure 2) and A549 co-cultures (Figure 6c). These results suggest that trogocytosis may be one of the preferred mechanisms of cell-to-cell transmission pathway for H5N1 viruses. However, in the present study, we observed that the H5N1 viral transmission rate in the A549 cells was slower than that in the MDCK cells. The slower rate of viral transmission in A549 co-culture cells is likely from slower kinetics of the H5N1 virus in A549 cells as compare to MDCK cells (Appendix A Figure A1a).

Unlike human influenza, replication of HPAI H5N1 viruses is highly efficient not only in respiratory epithelial cells but in many other cells. The HA with multibasic cleavage site of the HPAI virus may partially explain the phenomenon, but the mechanistic de-tails regulating the high replication of HPAI H5N1 are still incomplete. Trogocytosis is a form of cell–cell communication that can modulate the immune response and could be hijacking this existing intercellular network by pathogens [10,11,12,13,14,33]. We believed that the H5N1 virus might efficiently trigger some intracellular signaling or molecules in the infected cells, enhancing the trogocytosis. Although no direct evidence yet, we hypothesize that the multibasic cleavage site, HA protein of the H5N1 virus, interaction with its receptor α2,3 sialic acid at receptor tyrosine kinases during cell–cell contact in the co-culture may display the critical players required for triggering trogocytosis. In a previous report, the enrichment of phosphotyrosine at the cell–cell synapse led to increased trogocytosis [34]. Interestingly, Eierhoff et al. reported that the binding of viral HA to sialic acids at the receptor tyrosine kinases (RTK), such as epidermal growth factor receptor (EGFR), promotes the influenza virus uptake [35]. These receptor–ligand interactions lead to clustering of lipid rafts, which trigger receptor kinase activity resulting in signaling to the downstream pathway, such as the Ras/ERK/MAPK, PI3K/Akt, and JAK/STAT pathways [35]. The activation of EGFR also has stimulated the actin dynamics signaling pathway through RhoA, Rac1, and CDC42 pathways [36]. Side-by-Side comparisons of induction of trogocytosis by the HA with or without multibasic cleavage site should be further investigated**.**

Previously, TNTs have been well recognized as a component involved in cell-to-cell transmission in human influenza [16,17]. In this study, we proposed that the formation of TNT could be a consequence of the trogocytosis process. The live-cell images showed that TNTs were formed upon disassembly of the trogocytosis junction when the infected donor and recipient cells separated (Figure 4a). This finding was supported by studies of TNT formation between immune cells, which form an immunological synapse, which showed that a nanotube is formed as the cells dissociated [37,38,39,40,41].

Cell-to-cell transmission has been reported to have advantages for influenza virus infection under antiviral drug treatment [4,15]. In our experiment, we used oseltamivir, a NA inhibitor, to prevent the cell-free virus from spreading by inhibiting the virus release from the infected cells to demonstrate the evidence of cell-to-cell transmission of influenza viruses. Despite the use of oseltamivir as high as 100 μM to block cell-free transmission, we can still see the viruses transmitted to the recipient cells (Figure 5). This supports the existence of cell-to-cell transmission pathway and agrees with a previous study by Roberts, K.L. et al. that cell-to-cell transmission of influenza virus in the presence of the NA inhibitor was shown to occur by transport of vRNP to the adjacent cells through the intercellular connections [16]. However, our results showed that in the oseltamivir treated co-cultures have a much more pronounced effect on reducing virus transmission compared to those in the cytochalasin D. Disruption of the actin polymerization eventually affects on survival of the cells. The toxicity of cytochalasin D to the cell restricted the dose to less than 20 μM resulting in an incomplete block of viral spread via cell-to-cell transmission. These findings agree with other previous studies that cytochalasin D alone could inhibit the trogocytosis transfer, TNT formation, and virological synapses by only 50% [27,42,43]. Treatment with oseltamivir restricted the influenza virus to transmit by only cell-to-cell transmission pathway, which was mainly relied on actin polymerization [16]. Therefore, the combination of oseltamivir and cytochalasin D treatment led to a dramatic reduction of viral infection. Altogether, we confirmed that the H5N1 virus could transmit to the recipient cell via the cell-to-cell pathway, which resisted the effects of the NA inhibitor, and showed that this mode of transmission required actin polymerization as reported for the cell-to-cell transmission of the human influenza virus [15,17,22]. Although treatment with oseltamivir was inadequate to potently inhibit H5N1 cell-to-cell transmission, it remained important because virus transmission was significantly reduced in its presence (Figure 5). Cell-to-cell transmission of H5N1 was fast (Figure 2). As a result, early treatment is very important. It has previously been found that treating patients with oseltamivir within 48 h of symptom onset resulted in significantly increased patient survival [44,45]. In sum, our results suggested that cell-to-cell transmission provides a route for the H5N1 virus to escape and continue to spread during oseltamivir monotherapy. Moreover, combination therapy of oseltamivir and zanamivir showed no significant difference in the effectiveness compared to monotherapy [46,47], suggesting that the current antiviral drugs were not sufficient for influenza treatment. Thus, developing drugs targeting a cellular host factor to block cell-to-cell transmission pathways, besides targeting the virus directly, could be essential for developing new therapeutic strategies to control H5N1 infection. These inhibitors could be useful for use either alone or in combination with other current drugs. Although we described here how trogocytosis has an impact on the differences in viral infection rates, there are large gaps in our knowledge on the exact functions and roles of trogocytosis during virus infection. Hence, these roles need to be explored further. Many questions about the molecular mechanisms underlying the process of trogocytosis mediated by the H5N1 virus remain unclear. It will be interesting for future studies to identify the viral proteins and processes responsible for H5N1-induced trogocytosis. Additional investigations of cell-to-cell transmission and trogocytosis in other cell types, such as primary alveolar cell, endothelial cell, and immune cells, will shed light on the importance of this mode of transmission at time points as early as 10 min into H5N1 infection and provide insights on how H5N1 virus disseminates from the respiratory system and evades the immune response during systemic infection.

In conclusion, our results provided evidence demonstrating that trogocytosis could be the additional mechanism utilized by the influenza virus for cell-to-cell transmission in the MDCK and A549 cell lines. The H5N1 virus can use this means more effectively than the H1N1 virus, which likely contributes to the more severe pathogenesis of infection for the H5N1 virus. Thus, understanding the molecular basis of H5N1 cell-to-cell transmission and trogocytosis is urgently needed to progress our knowledge on H5N1 pathogenesis and to develop new antiviral strategies to inhibit this highly efficient mode of transmission such that improved treatment options will be available to rescue those who suffer from H5N1 infection.

## 4. Materials and Methods

### 4.1. Cell Line and Viruses

MDCK cells were cultured in MEM with 10% FBS, 100 U/mL penicillin, and 100 µg/mL streptomycin (Gibco, Grand Island, NY, USA). A549 cells were cultured in DMEM with 10% FBS, 100 U/mL penicillin, and 100 µg/mL streptomycin (Gibco). A highly pathogenic avian influenza (HPAI) H5N1 strain, A/open-billed stork/Nakhonsawan/BBD0104F/04 isolated from an Asian open-billed stork and a human influenza virus strain, A/Puerto Rico/8/34 were used. Viral stocks were propagated in MDCK cells. Briefly, the virus was adsorbed onto a monolayer of MDCK cells at 37 °C for 1 h. H5N1-infected cells were washed, and the infection media was replaced with serum-free MEM. For the H1N1 virus, the infection media was replaced with serum-free MEM containing 2 μg/mL of TPCK-treated trypsin. Infected cells were incubated at 37 °C with 5% CO_2_. Virus-containing supernatant was collected when a 3+ to 4+ cytopathic effect (CPE) was observed. Viral titers were determined by plaque assay. All procedures involving H5N1 virus were performed in a certified Biosafety level 3 laboratory at the Department of Microbiology, Faculty of Science, Mahidol University, Thailand.

### 4.2. Cell Membrane Labeling

The cell membrane of recipient cells was labeled with the fluorescent cell linker, PKH-26 Red (Sigma-Aldrich, St. Luis, MO, USA). MDCK and A549 cells were prepared at cell densities of 1 × 10^6^ cells in 250 μL of diluent C. The cell suspension was mixed with 250 μL of diluent C solution containing 1 μM PKH-26 dye and incubated at room temperature for 5 min. 500 μL of FBS was added to stop the labeling reaction for 1 min. Cells were washed twice with serum-free medium and resuspended with complete medium for use as recipient cells in further experiments.

### 4.3. Co-Culture and Transwell Assay

A monolayer of MDCK cells was infected with H5N1, or H1N1 viruses at an MOI of 1 for 8 h. Infected cells were trypsinized and used as donor cells. The donor cells (1 × 10^5^ cells) were mixed with PKH-26 labeled cells (recipient cells) (1 × 10^5^ cells) at a ratio of 1:1 in the culture media containing 2 μg/mL of TPCK-treated trypsin and cultured on a coverslip in a 24-well plate for direct co-cultures. For Transwell assays, 1 × 10^5^ donor cells were added onto a 0.4 µm polycarbonate Transwell insert (Corning, Corning, NY, USA). 1 × 10^5^ recipient cells were then added in the lower chamber at a ratio of 1:1. In some experiments, 100 µM of oseltamivir carboxylate (a potent inhibitor of NA) or 20 µM of cytochalasin D (F-actin inhibitor) (Sigma-Aldrich, St Luis, MO, USA) or combinations of these drugs were added into the culture medium. After 0, 1, 3, 6, 12, and 24 h of co-culture, cells were harvested and subjected to immunofluorescence assays. Cells were harvested after 0, 1, 2, and 3 h of co-culture for flow cytometry. The supernatant was collected to measure viral output by plaque assay. For co-culture assay in A549 cells, a monolayer of A549 cells was infected with H5N1 or H1N1 viruses at an MOI of 10 for 24 h. Infected cells were trypsinized and mixed with PKH-26 labeled cells (recipient cells) at a ratio of 1:1 in the culture media containing 2 μg/mL of TPCK-treated trypsin and cultured in a 24-well plate for direct co-cultures. Cells were then harvested after 0, 1, 3, and 6 h of co-culture for flow cytometry.

### 4.4. Live-Cell Imaging

A monolayer of MDCK cells was infected with H5N1 at an MOI of 1 for 8 h. Infected cells were trypsinized and labeled with PKH67 used as donor cells. The donor cells were mixed with PKH-26 labeled cells (recipient cells) at a ratio of 1:1 in 96-well plates. Cells were then imaged every 10 min for 6 h using the Operetta CLS imaging analysis system (PerkinElmer) in a temperature- and CO_2_-controlled chamber.

### 4.5. Immunofluorescence Assay

Cells cultured on the coverslip were washed with PBS, fixed with 4% paraformaldehyde for 1 h at room temperature and then washed three times with PBS. The cells were blocked with 1% bovine serum albumin (BSA) in phosphate buffer saline (PBS) for 1 h, washed and permeabilized with Cytofix/Cytoperm reagent (Becton Dickinson, Franklin Lakes, NJ, USA) for 10 min at 4 °C and then washed with PBS. To detect viral nucleoproteins (NP), cells were incubated with 1:100 FITC conjugated mouse anti-NP antibodies (Chemicon International) diluted in blocking buffer at room temperature for 1 h. Cells were rinsed with PBS, followed by deionized water, and then mounted in Prolong Gold antifade reagent containing DAPI counterstain (Invitrogen, Foster City, CA, USA). Stained cells were analyzed by fluorescence microscopy (Nikon, Tokyo, Japan). Cells from live-cell imaging were washed with PBS, fixed with 4% paraformaldehyde for 1 h at room temperature. Cells were then blocked with blocking buffer, washed, and permeabilized with 0.1% saponin containing blocking buffer. For F-actin staining, cells were incubated with 1:500 Alexa 647 Phalloidin (Thermo Fisher Scientific, Waltham, MA, USA). Cells were then analyzed by the Operetta HC imaging analysis system (PerkinElmer, Hamburg, Germany).

### 4.6. Flow Cytometry

Cells were washed and fixed with 4% paraformaldehyde at 4 °C for 30 min. Subsequently, cells were permeabilized in Cytofix/Cytoperm reagent (BD Biosciences, San Jose, CA, USA) at 4 °C for 10 min, then washed with perm/wash (BD Biosciences, San Jose, CA, USA). Cells were incubated with 1:500 FITC-conjugated anti-NP antibody (Millipore, Burlington, MA, USA) at 4 °C for 30 min, washed and suspended in 2% paraformaldehyde. Stained cells were detected using CytoFLEX flow cytometry (Beckman Coulter, Brea, CA, USA), and the data were analyzed using Kaluza Analysis software version 2.0. (Beckman Coulter, Brea, CA, USA).

### 4.7. Quantification of NP Gene Expression by Strand-Specific RT–qPCR

MDCK cells were infected with H5N1 virus at MOI of 1 for 8 h. Infected cells were trypsinized used as donor cells. The donor cells were mixed with recipient MDCK cells at a ratio of 1:1 in a 24-well plate for 0, 1, 2, and 3 h. Total RNA was extracted using the RNeasy mini kit (Qiagen, Hilden, Germany). The quantification of mRNA was performed by using strand-specific RT–qPCR with specific primers as described in [48]. Briefly, cDNA was reverse transcribed using AMV reverse transcriptase (Promega, Madison, WI, USA). Strand-specific RT primers used for the reverse transcription are NP mRNA RT (5′-CCA GAT CGT TCG AGT CGT TTT TTT TTT TTT TTT TCT TTA ATT GTC-3′); β-actin reverse (5′-AGG ATC TTC ATG AGG TAG TCA GTC AG-3′). Quantitative real-time PCR (qPCR) was performed using HotstarTaq DNA polymerase (Qiagen) contains fluorescent DNA dye SYBR Green. The following primers were used: NP mRNA forward (5′-CGA TCG TGC CCT CCT TTG-3′); NP mRNA reverse (5′-CCA GAT CGT TCG AGT CGT-3′); β-actin forward (5′-CCA CAC TGT GCC CAT CG-3′); β-actin reverse (5′-AGG ATC TTC ATG AGG TAG TCA GTC AG-3′). The cycle conditions of qPCR were 95 °C for 15 min, followed by 40 cycles of 95 °C for 30 s, 60 °C for 30 s and 72 °C for 30 s. The relative mRNA expression levels were determined by the 2(-delta delta C(T)) method using the housekeeping gene β-actin as a reference [49]. The mRNA fold changes of co-culture in each time point were compared to time point 0.

### 4.8. Plaque Assay

A confluent monolayer of MDCK cells was inoculated with a series of ten-fold dilution of influenza virus for 1 h. After the incubation, the supernatant was removed and overlaid with a plaque assay medium containing 0.6% Oxoid agar (Oxoid, UK). The plaque was incubated at 37 °C for 48 h. Thereafter, the monolayer was fixed with 3.7% formaldehyde for 1 h. The agar was removed, and the cell monolayer was stained with 1.25% crystal violet. Plaque forming unit per milliliter (pfu/mL) was calculated.
